# Direct Proteomic Detection and Prioritization of 19 Onchocerciasis Biomarker Candidates in Humans

**DOI:** 10.1016/j.mcpro.2022.100454

**Published:** 2022-11-23

**Authors:** Bruce A. Rosa, Kurt Curtis, Petra Erdmann Gilmore, John Martin, Qiang Zhang, Robert Sprung, Gary J. Weil, R. Reid Townsend, Peter U. Fischer, Makedonka Mitreva

**Affiliations:** 1 Division of Infectious Diseases, Department of Medicine, Washington University School of Medicine, St Louis, Missouri, USA; 2 Division of Endocrinology, Metabolism and Lipid Research, Department of Medicine, Washington University School of Medicine, St Louis, Missouri, USA; 3 Department of Genetics, Washington University School of Medicine, St Louis, Missouri, USA; 4 McDonnell Genome Institute, Washington University School of Medicine, St Louis, Missouri, USA

**Keywords:** onchocerciasis, biomarker, proteomics, bioinformatics, OV-pos, Samples from individuals infected with *Onchocerca volvulus*, OV-neg, Samples from uninfected individuals, PSM, Peptide-spectral matches, MDA, Mass drug administration, DTT, Dithiothreitol, FASP, Filter-aided sample preparation, TFA, Trifluoroacetic acid, PASEF, Parallel accumulation-serial fragmentation

## Abstract

*Onchocerca volvulus*, the causative agent of onchocerciasis, infects over 20 million people and can cause severe dermatitis and ocular conditions including blindness. Current treatments employed in mass drug administration programs do not kill adult female worms, and common diagnostic tests cannot reliably assess viability of adult worms. There is an urgent need for better diagnostic tests to facilitate monitoring the efficacy of new treatments and disease elimination efforts. Here, eight plasma samples collected from individuals infected with *O. volvulus* and seven from uninfected individuals were analyzed by MS/MS spectrometry to directly identify *O. volvulus* proteins present in infected but absent in uninfected control samples. This direct proteomic approach for biomarker discovery had not been previously employed for onchocerciasis. Among all detected proteins, 19 biomarker candidates were supported by two or more unique peptides, identified in the plasma of at least three *O. volvulus*-infected human samples and absent in all control samples. Comprehensive analysis and ranking of these candidates included detailed functional annotation and a review of RNA-seq gene expression profiles. Isotope-labeled standard peptides were run in parallel and validated MS/MS peptide identifications for 15 peptides from 11 of the 19 proteins, and two infected urine and one uninfected urine sample was used for additional validation. A major antigen/OVOC11613 was identified as the most promising candidate with eight unique peptides across five plasma samples and one urine sample. Additional strong candidates included OVOC1523/ATP synthase, OVOC247/laminin and OVOC11626/PLK5, and along with OVOC11613, and were also detected in urine samples from onchocerciasis patients. This study has identified a promising novel set of proteins that will be carried forward to develop assays that can be used for diagnosis of *O. volvulus* infections and for monitoring treatment efficacy.

## Highlights

• MS proteomics was performed directly on plasma and urine samples
• Matched negative controls were used to reduce false positives
• 19 novel *O. volvulus* biomarker candidates were identified and prioritized
• Candidates were validated by isotope-labeled heavy peptides

## Graphical Abstract

**Figure:**
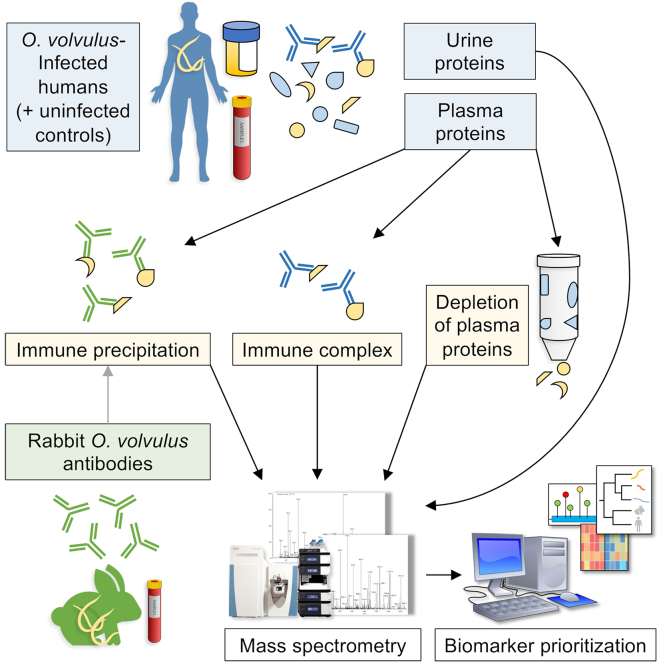

## In Brief

*Onchocerca volvulus* infects over 20 million people, causing severe disability. Diagnostic tests cannot reliably detect live adult worms, which is critical for disease elimination efforts. Plasma and urine samples from *O. volvulus*-infected individuals (and uninfected controls) were analyzed by MS proteomics to directly identify *O. volvulus* proteins in hosts. A total of 19 proteins were prioritized. This set of biomarker candidates is a valuable resource to further explore for the diagnosis of active *O. volvulus* infections.

*Onchocerca volvulus* is a parasitic nematode transmitted by *Simulium* blackflies and is the causative agent of onchocerciasis, which is also known as “river blindness”. This debilitating neglected tropical disease can cause severe dermatitis and a variety of ocular conditions that can lead to visual impairment and permanent blindness (1). As of 2017, 20.9 million people were estimated to be infected, with 14.6 million afflicted with skin disease and 1.2 million with vision loss (2). Onchocerciasis is currently targeted for elimination, and in 2020,112 million people in Africa received ivermectin by mass drug administration (MDA) of a total of 239 million people who require MDA (1, 3).

While ivermectin clears first stage larval parasites (microfilariae, Mf) from the skin and eyes, adult *O. volvulus* female worms are not killed by this treatment. Mf repopulate the skin a few months after ivermectin treatment, so repeated rounds of MDA are required to reduce Mf in the skin and reduce transmission of infective larvae (4). There is an urgent need for treatments that effectively kill or permanently sterilize adult female worms. The efficacy of triple drug combination therapy with ivermectin, diethylcarbamazine, and albendazole (IDA), which kills or sterilizes other filarial parasites for onchocerciasis is unknown (5). Diagnostic tests commonly used to detect the Mf such as skin snips, diethylcarbamazine patch tests, and PCR cannot reliably assess the viability of adult worms. Adult worm viability can be assessed by microscopic examination of surgically removed onchocercal nodules, but this is invasive and expensive, and it requires special expertise. Antibody tests for onchocerciasis such as those based on recombinant antigen Ov16 (6, 7) are not reliable markers for the presence of viable adult worms, because antibodies persist long after the death of adult worms (8, 9). This makes it difficult to use this test to inform decisions about whether MDA should be continued (10). Other studies have identified additional antigen candidates, including OVOC3261 (11) and other *O. volvulus* proteins, that are specifically bound by IgG from infected individuals that are *O. volvulus*-specific and highly expressed by adult female worms (12). While these antigens may improve sensitivity, it is not clear that any antibody assay will prove to be specific for current infection. Thus, there is an urgent need for better diagnostic tests for onchocerciasis to detect active infections and to monitor the efficacy of new treatments (13).

Problems with antibody serology have led to attempts to detect worm products or metabolites in bodily fluids. Early claims of success (for example with *N*-acetyltyramine-*O*,β-glucuronide (14)) were not independently confirmed for sensitivity or specificity (15, 16). Another metabolomics study has identified two potential plasma biomarkers for *O. volvulus* infection (inosine and hypoxanthine) which had generally low detection levels in a separate nonendemic cohort but have yet to be validated in a separate cohort of infected individuals (17). Phospholipid profiling of *O. volvulus*, *Onchocerca ochengi*, and *Litomosoides sigmodontis* identified species-specific lipids, but while the lipid biomarker phosphatidylethanolamine was detected at high levels in *O. ochengi* nodules in cows, the potential plasma or serum lipid biomarkers were below detection limits in infected animals and humans (18). Additionally, miRNA profiling study identified four *O. volvulus*-derived mature miRNAs in the serum and plasma of infected individuals and not in uninfected individuals, with two (miR-71 and lin-4) being detected in individuals from both Ghana and Cameroon (19). At present no commercially available test is sensitive or specific for detection of active infections by adult worms.

In this study, we have generated new mass-spectrometry data from *O. volvulus* infected and uninfected subjects and performed a multiplatform analysis of proteomic, genomic, and transcriptomic *O. volvulus* datasets that resulted in identification, characterization, and prioritization of *O. volvulus* candidate biomarkers that could be used to develop assays for diagnosis of adult females. These candidate biomarkers have been identified by proteomic detection in the blood and urine of several infected individuals and not in uninfected individuals, and additional proteomic validation is provided by the manual evaluation of spiked-in heavy peptides. The long-term goal of this initiative is to further evaluate these promising biomarker candidates for their use to detect active infections and monitor treatment efficacy.

## Experimental Procedures

### Experimental Design and Statistical Rationale

A total of 18 independent biological replicates were analyzed in the MS/MS proteomics analysis: Plasma and urine samples collected from individuals infected with *O. volvulus* (OV-pos; eight plasma replicates, two urine replicates) and from uninfected individuals used as negative controls for detection (OV-neg; seven plasma replicates, one urine replicate). Some samples were collected from individuals and some represented pooled samples as indicated in supplemental Table S1. Because data collection for this project has spanned many years, several different LC:MS technologies were used for data collection, including hybrid quadrupole Orbitrap (Q-Exactive) and trapped ion mobility time-of-flight (timsTOF) mass spectrometers. Technical replicates were collected from some samples from two different MS/MS proteomics machines (Q Exactive or timsTOF), as indicated per sample in supplemental Table S1. Fractionation of timsTOF samples was also performed, with separate runs per sample. For all technical replicates (including multiple fractions of the same sample), peptide-spectral matches (PSMs) were summed for each peptide in each sample, and they were not considered as replicates for any statistical analyses or presented as replicates in any figures or tables. A stepwise filtering process based on presence/absence across OV-pos and OV-neg samples is described in detail in the Results/Discussion and did not rely on any statistical test validation. The overlap between some identified gene sets of interest with previous results including functional annotations and RNA-seq data were tested using a Fischer exact test or a two-tailed *t* test (unequal variance). Informed consent was provided by the patients in accordance with the Declaration of Helsinki.

### Plasma and Urine Sample Collection

Origins, collection dates, and sample processing data for all samples analyzed by proteomics are summarized in supplemental Table S1. Each plasma sample contained either an individual plasma sample or a pooled sample with up to 15 plasma samples, containing equal volumes of each patient sample (usually 100–250 μl plasma each). Samples from subjects infected with *O. volvulus* were collected in Uganda (20) or Cameroon (21), and Mf density was determined by duplicate skin snips as described previously (20). Plasma samples from Uganda were from an area hyperendemic for onchocerciasis before any intervention. All donors had palpable nodules and high skin Mf densities over 50 Mf/mg (see supplemental Table S1). Samples from Cameroon were from subjects with low to moderate Mf densities. Additional samples from uninfected individuals were collected from India (22) and from the USA. No samples were collected from lymphatic filariasis–endemic areas. Ugandan samples were collected from an area co-endemic for *Mansonella perstans*, but all tested samples were negative for *Mansonella* Mf by a modified Knott technique. *Wuchereria bancrofti* is not endemic most parts of Cameroon, and blood samples collected during the day were negative for *Loa loa* and *M. perstans* Mf. Onchocerciasis is not endemic to India, and blood samples were negative for circulating *W. bancrofti* antigen. The uninfected control samples from the USA were collected from uninfected volunteers from Barnes Jewish Hospital and represent serum samples, but for the sake of consistency in labeling, are labeled here as plasma samples. For these samples, nonendemic control sera samples were obtained de-identified from the Barnes-Jewish-Christian Hospital Clinical laboratory in St Louis. The Washington University in St Louis Human Research Protection Office (an institutional review board) determined that work with these de-identified samples did not constitute human subjects research. In addition, the use of human samples for the development of new diagnostics was approved by the IRB at the Washington University School of Medicine under the ID #201102546.

Plasma samples were collected in EDTA-coated tubes and stored in 1 ml aliquots in cryovials (Nunc, Thermo Fisher Scientific). Plasma samples collected in filarial parasite endemic countries underwent at least one freeze-thaw cycle, but not more than three freeze-thaw cycles. Plasma samples were stored long term without specific protease inhibitors at −80 °C. Urine samples were treated similarly, but aliquots were stored in 5 ml tubes.

Samples were assessed by LC-MS following (i) depletion to enrich for potential target proteins by removal of common human plasma proteins, (ii) immunoprecipitation (IP) using antiparasite antibodies, or (iii) by precipitation of immune complexes (IC). All of these approaches are detailed below. Urine samples were exclusively individual samples and were concentrated but were not depleted for host proteins.

### IgG and IP Preparation

Rabbits were immunized with *O. volvulus* adult worm extract, and IgG was prepared from serum by ammonium sulfate precipitation as previously described (23). Total IgG was isolated from plasma samples by ammonium sulfate (Sigma-Aldrich) precipitation. Ammonium sulfate was added dropwise, with constant sample mixing for a final concentration of 50%. The mixture was incubated overnight at 4 °C causing IgG precipitation and then centrifuged at 3000*g* for 30 min at 4 °C. The IgG pellet was suspended in PBS and dialyzed extensively against PBS (Thermo Fisher Scientific) using 20 kDa MWCO membrane cassette (Thermo Fisher Scientific) to remove ammonium sulfate. IgG samples were coupled to Pierce NHS-Active Agarose (Thermo Fisher Scientific). For 1 ml of IgG-bound agarose, 2 ml of 50% NHS-Active Agarose was added to a 2 ml Pierce centrifuge column (Thermo Fisher Scientific), and the column was centrifuged at 1000*g* for 1 min in a swinging bucket rotor to remove the column buffer. The agarose was washed by adding 2 ml of ultra-pure water to the column and centrifuging at 1000*g* for 1 min. Two milliliter IgG in PBS (20 mg IgG) was added to the agarose and incubated for 2 h at room temperature with constant mixing, then it was centrifuged at 1000*g* for 1 min. Two milliliter PBS was then added to the column as a wash and centrifuged at 1000*g* for 1 min. The column was then blocked using 2 ml 1.0 M ethanolamine (pH 7.4; Sigma-Aldrich), incubated for 20 min at room temperature, and centrifuged at 1000*g* for 1 min. Six milliliter PBS was added to the column and centrifuged at 1000*g* for 1 min as a final wash. The final 1 ml of beads were then diluted in PBS to a final volume of 10 ml (10% working dilution for IP).

Rabbit anti-*O. volvulus* IgG beads were used to capture *O. volvulus* antigens in human sera. Human plasma (250 μl) was added to an equal volume of 10% IgG beads in a 1.5 ml microcentrifuge tube (Sigma Aldrich) and incubated overnight at 4 °C. The sample was centrifuged 12,400*g* for 2 min, the supernatant was discarded, and the pelleted beads were washed three times in PBS with Tween (Thermo Fisher Scientific).

### Immune Complex Preparation

Human plasma (400 μl) was added to an equal volume of 0.2 M EDTA pH 7.5 and 12% PEG 8000 (Thermo Fisher Scientific) in 0.1 M borate buffer pH 8.3. The mixture was incubated overnight at 4 °C and then centrifuged at 16,000*g* for 30 min. The supernatant was discarded, and pellet was washed three times with ice-cold 2.4% PEG 8000 in 0.1 M borate buffer pH 8.3. The final pellet was then submitted for MS analysis.

### Multiaffinity Fractionation of Plasma

Fourteen of the highest abundant plasma proteins (albumin, IgG, α1-antitrypsin, IgA, IgM, transferrin, haptoglobin, α2-macroglobulin, fibrinogen, complement C3, α1-acid glycoprotein [orosomucoid], HDL [apolipoproteins A-I and A-II], and LDL [mainly apolipoprotein B]) were removed (>95%) using Seppro LC5IgY14 columns (Sigma Aldrich) according to vendor protocol.

Processed plasma samples as described above were thawed and centrifuged at 15,000*g* for 10 min. The supernatants were transferred to a 1.5 ml tube and an equal volume of 2× Tris buffered saline (300 mM NaCl, 20 mM Tris-HCl, pH 7.4) was added. The diluted supernatant was filtered with a 0.45 μm spin filter and centrifuged at 10,000*g* for 10 min. A 25 μl aliquot of the concentrate was transferred to a 0.5 ml tube and 85 μl TBS (150 mM NaCl, 10 mM Tris-HCl, pH 7.4) was added. Then, 110 μl of sample was transferred to an autosampler vial and kept at 4 °C. Multiaffinity removal of high-abundance proteins was performed using an Agilent 1260 Infinity liquid chromatography system with an Agilent Hu-14 column, 4.6 mm × 50 mm. The samples (0.1 ml) were loaded onto the column at a flow rate of 0.5 ml/min. The column effluent was monitored at *A*_280._ The flowthrough fractions (2.25 ml) were collected in TBS. The bound fractions (4.25 ml) were eluted with 0.1 M glycine, pH 2.5. The flow through and bound fractions were concentrated using a 3000 MWCO spin filter. For deep-scale proteomic analysis runs, multiple aliquots were prepared using multiaffinity chromatography as described above and transferred into separate 3K filter units. Protein concentration was determined by a BCA assay before freezing samples.

Protein was prepared from urine (15 ml) by concentration using a 5000 MWCO K filter to ∼200 μl. The concentrated urine was precipitated with cold acetone (1:5 parts of sample to cold acetone (vol/vol)). The samples in cold acetone were kept at −20 °C overnight followed by centrifugation for 10 min at 20,000*g*. The protein pellets were solubilized in SDS buffer with sonication, and protein concentration was determined by BCA assay. IC pellets were solubilized in SDS buffer with sonication, and protein concentration was determined by BCA assay. Immunoprecipitated proteins were eluted from antibody beads with 0.1 M glycine (pH 2) followed by solubilization with SDS buffer. The eluates were lyophilized and digested as described below using filter-aided sample preparation methods (FASP).

### Preparation of Peptides

A summary of peptide preparation methods for the each of the samples is provided in supplemental Table S1. Peptides were prepared from multi-affinity fractionated sera by endoprotease digestion of ∼250 μg of the “depleted” sera. The buffer (100 mM Tris-HCl, pH 8.0) containing 10 M urea was added to the “depleted” samples to a final concentration of 8 M urea. Disulfide bonds were reduced with 6.5 mM dithiothreitol (Pierce Chemical) for 45 min at room temperature and the Cys residues were alkylated with 13 mM iodoacetamide (Pierce Chemical) for 45 min at room temperature in the dark. The urea concentration was diluted to 2 M by addition of 100 mM Tris-HCl buffer (pH 8.0) prior to addition of 5 μg of LysC (Wako Chemicals). Samples were incubated at room temperature for 2 h, followed by addition of trypsin (1:100 enzyme/substrate) and digestion overnight at room temperature. Peptides were desalted on a 200 mg Sep-PAK column. Peptides were eluted with 1.5 ml 50% (vol/vol) MeCN, 0.1% (vol/vol) FA. An aliquot (10%) of the eluate was assayed for peptide quantity, and the remainder was lyophilized and stored at −80 °C.

The IC pellets were solubilized with 30 μl of SDT buffer (4% [wt/vol], 100 mM Tris-HCl pH 8.0). Protein disulfide bonds were reduced using 100 mM DTT with heating to 95 ºC for 10 min. Peptides were prepared as previously described using a modification (24) of the FASP (25). The samples were mixed with 200 μl of 100 mM Tris-HCL buffer, pH 8.5 containing 8 M urea (UA buffer). The samples were transferred to the top chamber of a 30,000 MWCO cutoff filtration unit (Millipore) and processed to peptides as previously described (25). The peptides were dried in a Speedvac concentrator (Thermo Fisher Scientific, Savant DNA 120 Speedvac Concentrator) for 15 min. The dried peptides were dissolved in 1% (vol/vol) TFA and desalted using two microtips (porous graphite carbon, BIOMEKNT3CAR) (Glygen) on a Beckman robot (Biomek NX), as previously described (Chen Mol. Cell Proteomics PMID 22338125). The peptides were eluted with 60 μl of 60% (vol/vol) MeCN in 0.1% (vol/vol) TFA and dried in a Speed-Vac (Thermo Fisher Scientific, Model No. Savant DNA 120 concentrator) after adding TFA to 5% (vol/vol). The peptides were dissolved in 20 μl of 1% (vol/vol) MeCN in water. An aliquot (10%) was removed for quantification using the Pierce Quantitative Fluorometric Peptide Assay kit (Thermo Fisher Scientific, Cat. No. 23290). The remaining peptides were transferred to autosampler vials (Sun-Sri, Cat. No. 200046), dried, and stored at −80 °C. For samples treated with PNGaseF before digestion, samples were exchanged into ammonium bicarbonate buffer (100 mM, pH 8.0) after alkylation, then incubated with 20 U PNGaseF in 100 μl of the buffer for 1 h in a thermomixer at 600 rpm. Protein digestion and peptide preparation were then performed using the FASP method.

### Off-Line Fractionation of Peptides

The peptides prepared from depleted sera, ICs, and urine were fractionated using basic pH reverse-phase chromatography prior to LC-MS (26). The chromatograph was an Agilent HPLC system 1260 equipped with an Agilent Zorbax 300Extend-C18, 4.6 × 250 mm column. The peptides were separated using a binary gradient of solvent A (4.5 mM ammonium formate (pH 10) in 2% (vol/vol) MeCN) and solvent B (4.5 mM ammonium formate pH 10 in 90% (vol/vol) MeCN) at a flow rate of 1 ml/min. The gradient for equilibrating the column was as follows (time in min): %B: 0,0; 4,0; 19,100; 21,100; 22,0; 24,0; 39,100; 41,100; 42,0; 70,0.

The gradient method for peptide chromatography was as follows (time in min.): %B: 0, 0; 9, 0; 13, 6; 63, 28.5; 68.5, 34; 81.5, 60; 98.1, 60; 100, 0; 120, 0. The lyophilized peptides were dissolved in 465 μl of 4.5 mM ammonium formate (pH 10) in 2% (vol/vol) MeCN by vortexing. Each sample was centrifuged at 18,000*g* at RT for 5 min and transferred to the autosampler vial. The sample (450 μl) was loaded into the sample loop and injected onto the column for 1.2 min at a flow rate of 1 ml/min, and the gradient program was initiated. Column effluent was monitored at 214 nm. The column flow through and 96 fractions were collected. The 96 fractions were concatenated to 24 as previously described (26). The fractions were lyophilized to complete dryness (∼16 h). Gradient optimization and system monitoring was performed using standard of bovine serum albumin tryptic peptides (500 μg/analysis). System performance was evaluated prior to each sample analysis by comparison with legacy peak resolution and retention times from previously optimized chromatographies using standard peptides.

### Mass Spectrometry

The purified peptides from depleted plasma, ICs, and urine were analyzed using ultraperformance HPLC mass spectrometry. Initial studies were performed with a hybrid quadrupole Orbitrap instrument and more recently with a trapped ion mobility timsTOF (27).

For ultraperformance HPLC hybrid quadrupole Orbitrap mass spectrometry (28) the peptides in FA (1% (vol/vol)) were loaded in 2.5 μl onto a 75 μm i.d. × 50 cm Acclaim PepMap 100 C18 RSLC column (Thermo Fisher Scientific) on an EASY *nano*LC (Thermo Fisher Scientific) at a constant pressure of 700 bar at 100% A (0.1%FA). Prior to sample loading, the column was equilibrated to 100%A for a total of 11 μl at 700 bar pressure. Peptide chromatography was initiated with mobile phase A (1% FA) containing 2%B (100%ACN, 1%FA) for 5 min, then increased to 20% B over 100 min, to 32% B over 20 min, to 95% B over 1 min and held at 95% B for 19 min, with a flow rate of 250 nl/min. The data were acquired in data-dependent acquisition mode. The full-scan mass spectra were acquired with the Orbitrap mass analyzer with a scan range of *m/z* = 325 to 1500 and a mass resolving power set to 70,000. Ten data-dependent high-energy collisional dissociations were performed with a mass resolving power set to 17,500, a fixed lower value of *m/z* 100, an isolation width of 2 Da, and a normalized collision energy setting of 27. The maximum injection time was 60 ms for parent-ion analysis and product-ion analysis. The target ions that were selected for MS/MS were dynamically excluded for 20 s. The automatic gain control was set at a target value of 1e6 ions for full MS scans and 1e5 ions for MS2. Peptide ions with charge states of one or >8 were excluded for MS2 acquisition. QE parameter setting details are provided in supplemental Table S2*A*, and EASYnLC parameters are provided in supplemental Table S2*B*.

For timsTOF LC-MS, the peptides were separated using a *nano-ELUTE* chromatograph (Bruker Daltonics) interfaced to a timsTOF Pro mass spectrometer (Bruker Daltonics) with a modified nanoelectrospray source (CaptiveSpray, Bruker Daltonics). The mass spectrometer was operated in parallel accumulation-serial fragmentation (PASEF) mode (27). The samples in 2 μl of 1% (vol/vol) FA were injected at a flow rate of 400 nl/min onto the column (75 μm i.d. × 25 cm Aurora Series) with a CSI emitter (Ionopticks). The column temperature was set at 50 °C. The column was equilibrated using constant pressure (800 bar) with eight column volumes of solvent A (0.1% (vol/vol) FA). Sample loading was performed at constant pressure (800 bar) in 2 μl of solvent A.

The peptides were eluted using the following gradient of solvent B (0.1% (vol/vol) FA/MeCN): solvent A containing 2% B was increased to 17% B over 60 min, to 25% B over 30 min, to 37% B over 10 min, to 80% B over 10 min and constant 80% B for 10 min. The MS1 and MS2 spectra were recorded from *m/z* 100 to 1700. Suitable precursor ions for PASEF-MS/MS were selected from TIMS-MS survey scans by a PASEF scheduling algorithm (27). A polygon filter was applied to the *m/z* and ion mobility plane to select features most likely to be multiply charged peptide precursor ions. Quadrupole isolation width was set to two Th for *m*/*z* < 700 and three Th for *m*/*z* > 700. The collision energy was ramped stepwise as a function of increasing ion mobility: 52 eV for 0 to 19% of the ramp time; 47 eV from 19 to 38%; 42 eV from 38 to 57%; 37 eV from 57 to 76%; and 32 eV for the remainder. The TIMS elution voltage was calibrated linearly using the Agilent ESI-L Tuning Mix (*m/z* 622, 922, 1222). Detailed timsTOF parameters are provided in supplemental Table S2*C*, and Nanoelute parameters are provided in supplemental Table S2*D*.

### Peptide Detection From MS/MS Spectra

Detected MS/MS proteomics spectra were assigned to *O. volvulus* peptides using MaxQuant (29) (version 1.6.17), by searching against a database of proteins based on the current *O. volvulus* genome (30) annotation (PRJEB513, WBPS15, WS276; 12,224 proteins) and the “common Repository of Adventitious Proteins” database (cRAP, v2012.01.01, https://www.thegpm.org/crap/; 115 proteins) to screen for common contaminants. The searches were performed with a fragment ion mass tolerance of 40 ppm and a parent ion tolerance of 20 ppm. The enzyme search specificity was selected as tryptic/P, allowing for a maximum of four missed cleavages. Carbamidomethylation of cysteine residues was specified as a fixed modification. The searches were conducted with the following variable modifications: deamidation of asparagine, deamidation of glutamine, formation of pyro-glutamic acid from N-terminal glutamine, acetylation of protein N terminus, oxidation of methionine, and pyro-carbamidomethylation of N-terminal cysteine residues. Peptides and protein results were filtered at 1% false discovery rate by searching against a reversed protein sequence database. MaxQuant settings used for spectra-peptide assignments were defined in the xml configuration file, which is provided in supplemental Table S2*E*, and all MaxQuant output files are provided as part of the raw data upload to the iProX database (31) (IPX0004317000, ProteomeXchange Consortium identifier PXD033659; accession numbers provided in supplemental Table S1).

Resulting identified peptides were screened against the *Homo sapiens* proteome (GRCh38 release 105 (32) and including all isoforms; 117,909 isoform sequences from 20,465 coding proteins), treating leucine and isoleucine amino acids as matches since they are indistinguishable in the spectral matching. Any peptides exactly matching human sequences were discarded for all downstream analysis. Peptides were quantified per sample by the number of PSMs (supplemental Table S3*A*), but PSM counts were not utilized for any peptide filtering or statistical analysis. All 22,648 peptides assigned to *O. volvulus* sequences were also screened against *Wolbachia* (*Wolbachia* endosymbiont of *O. volvulus* str. Cameroon, NCBI tax id:1,410,384, GenBank release 250), and only four peptides had an exact match to Wolbachia, all of which were only detected in the whole-worm lysate sample. Those peptides are indicated with an asterisk in supplemental Table S3*A*. All data for *O. volvulus*-assigned spectral-peptide matches are provided in supplemental Table S3*B*, including charges, masses, modifications, and scores.

The number of peptides per protein per sample are provided as part of supplemental Table S4. Although all peptides matching all proteins are reported in the supplemental tables, only proteins supported by two or more unique peptides (as indicated in supplemental Table S4) were considered to be reliably detected in the absence of the detailed annotation of spectra, which can be performed using the available raw data (iProX database (31) accession IPX0004317000, ProteomeXchange Consortium identifier PXD03365).

**Fig. 1:**
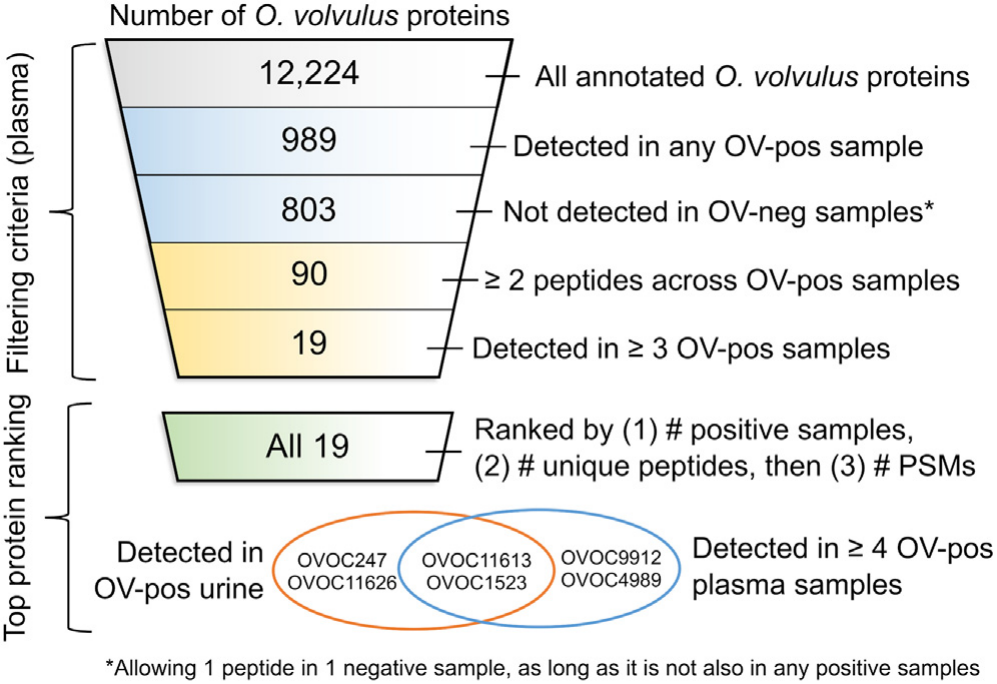
**Overview of the stepwise filtering and ranking process for the identification of potential *O. volvulus* biomarkers among human plasma proteomics samples.** PSM, peptide-spectral matches.

A stepwise filtering and ranking process for detected proteins was performed to identify those with the highest potential to serve as biomarkers for the presence of viable adult *O. volvulus* females. This analysis compared results obtained with onchocerciasis samples to those obtained with negative control samples as described in detail in the Results/Discussion section and in Fig. 1. Proteins identified at each step in the filtering and ranking process are indicated in supplemental Table S4.

### Transcriptome Sample Collection and Analysis

Unprocessed RNA-seq reads were retrieved from analyses described in a previous study (from eight adult female samples (12)). RNA-seq reads were mapped to the *O. volvulus* genome (30) (PRJEB513, WBPS15, WS276) using STAR (33), quantified with featureCounts (34), and normalized using DESeq2 (35). Relative expression was calculated using fragments per kilobase per million reads normalization per gene per sample. All normalized expression values are provided in supplemental Table S4.

### O. volvulus Deduced Protein Annotation Database

The *O. volvulus* genome/proteome annotation was downloaded from WormBase Parasite (36) (PRJEB513, version WBPS15 WS276). The retrieved functional annotations were complemented with additional assignments using InterProScan v5.42 (37) to identify gene ontology (38) classifications and InterPro functional domains (39) and GhostKOALA v2.2 (40) to assign KEGG (41) annotations. Potentially secreted proteins were identified using both SignalP v5.0 (42) for signal peptides and transmembrane domains and SecretomeP v2.0 (43) to identify proteins with nonclassical secretion sequences (where any proteins with two or more transmembrane domains were not classified as secreted).

Protein conservation data across nematodes and hosts were quantified using BLAST (44) and OrthoFinder (v2.4.1) (45), used to compare protein sequences across all species, and define orthologous protein families, using default values. Protein sequence data was downloaded from WormBase Parasite (36) (WBPS15 WS276) for *Ancylostoma ceylanicum* (PRJNA72583), *L. loa* (PRJNA246086), *Necator americanus* (PRJNA72135), *Ascaris lumbricoides* (PRJEB4950), *Caenorhabditis elegans* (PRJNA13758), *Brugia malayi* (PRJNA10729), *Trichuris trichiura* (PRJEB535), *Strongyloides stercoralis* (PRJEB528), *O. ochengi* (PRJEB1465), and *W. bancrofti* (PRJNA275548; improved annotation). In addition, host outgroups were downloaded from Ensembl (32) for *Oryctolagus cuniculus* (Rabbit; OryCun2.0), *H. sapiens* (Human; GRCh38.p13), and *Drosophila melanogaster* (BDGP6.32). The top BLAST (44) hit for each *O. volvulus* protein was identified, including the E value, alignment length, % identity, and whether the top hit was reciprocal (NCBI blastp v2.7.1+, default settings).

All protein functional annotations and orthology information including OrthoFinder groups and BLAST results for all *O. volvulus* proteins are provided in supplemental Table S4.

### Heavy Peptide Spectral Matching for Detected Peptide Validation

Isotope-labeled standard peptides (“heavy peptides”) were synthesized (Vivitide) for peptides shorter than 18 amino acids that were detected from the top 19 proteins from the proteomics analysis. These isotope-labeled heavy peptides were spiked into an aliquot of the sample where the candidate peptide was initially observed. supplemental Table S5 shows the observed *m/z* values, intensities, and mass error for the fragmentation ions from candidate biomarker peptides and complimentary values for the corresponding stable isotope labeled peptides which were used to corroborate peptide sequence assignment. The fragment ion mass tolerance for matching used was in agreement within ± 20 ppm and ± 40 ppm for the parent and fragment ions, respectively. Given the expected low abundance of *O. volvulus* proteins in blood and the potential for heterogeneous antibody responses across individuals, we anticipated that spectra for candidate biomarkers would be of low intensity and contain ions below the limit of detection, requiring manual interpretation. The minimum criteria for spectral matching/verification were agreement of the parent ion m/z value with the calculated value within mass error specification and observation of a series of fragment ions that defined a sequential amino acid sequence of four residues. Where only spectra from the modified peptide were observed, both the calculated values and the spectra from the synthetic, unmodified peptide were used for interpretation.

## Results and Discussion

To identify candidate biomarkers with potential to detect adult *O. volvulus* females, we performed mass spectrometry (MS) analyses with human plasma and urine samples from infected and uninfected individuals. False positive identification of biomarker candidates (a common problem in MS studies) was minimized by comparing MS results from eight individual or pooled plasma samples from infected people with results obtained from seven individual or pooled samples from uninfected people. Peptides or proteins were excluded as infection-detection candidates if they were also detected in negative samples. Analysis of three types of samples derived from plasma (ICs or IC, proteins immunoprecipitated from human samples with antibodies to *O. volvulus* or IP, and samples depleted of common plasma proteins; “depleted plasma”) increased chances for detecting signals. Detection in multiple positive samples prepared by different methods further increased confidence in the results. We also analyzed concentrated urine samples from infected and uninfected individuals to assess the potential of urine (with much less host protein than plasma) as an alternative to plasma for biomarker detection, and for additional confirmation for candidate proteins. Samples for the study were from Uganda and Cameroon (infected and uninfected samples), India, Indonesia, and the USA (uninfected samples; Table 1). This approach of using MS proteomics to directly screen both infected and uninfected human plasma and urine samples has not been previously reported for onchocerciasis.

**Table 1:** Overview of mass spectrometry proteomics sample sets used as part of the candidate biomarker filtering and ranking process. “OV-pos” = sample from individual(s) infected with *O. volvulus*; “OV-neg” = sample from individual(s) not infected with *O. volvulus*. “Ind.” = sample from single individual, “Pool” = sample from a pool of individuals

Sample group	Plasma treatment	Pooled/Individual	Country	Number of PSMs	Number of peptides	Number of proteins
OV-pos plasma samples	Depleted (Dep)	Pool	Uganda	220	121	116
		Pool	Uganda	439	190	181
		Pool	Uganda	565	252	241
	Immunoprecipitated (IP)	Pool	Uganda	370	211	203
	Immune complex (IC)	Ind.	Uganda	495	283	235
		Pool	Uganda	337	97	88
		Pool	Uganda	295	134	133
		Ind.	Cameroon	199	130	129
OV-pos urine	−	Ind.	Cameroon	266	163	156
	−	Ind.	Cameroon	104	75	73
OV-neg plasma samples	Depleted (Dep)	Pool	India	181	103	97
	Immunoprecipitated (IP)	Pool	Uganda	186	115	113
	Immune complex (IC)	Pool	India	172	98	95
		Ind.	India	91	72	68
		Ind.	India	65	55	52
		Ind.	India	36	31	30
		Pool	Indonesia	242	148	144
OV-neg Urine	−	Ind.	Cameroon	90	62	59
Worm lysate	−	−	−	70,226	21,109	2405

### Production of a Comprehensive Dataset to Support Biomarker Identification and Characterization

Plasma and urine samples collected from OV-pos individuals (eight plasma, two urine) and from uninfected individuals (OV-neg; seven plasma, one urine) were analyzed by MS/MS proteomics. Several proteomic workflows were used to prepare peptides for LC/MS analysis, including from plasma with 14 high abundance proteins removed, immunoprecipitates, and ICs. Details of sample processing and MS configuration are provided in the materials and methods and sample metadata, raw data accessions (from the iProX database (31) accession IPX0004317000, ProteomeXchange Consortium identifier PXD033659), and processing information are provided in supplemental Table S1. Mass spectrometric configuration data for all machines are provided in supplemental Table S2, *A*–*D*.

For all datasets, MaxQuant (29) was used to match MS/MS spectra to *O. volvulus* peptides, using the same settings and approach for all samples (configuration file provided in supplemental Table S2*E*). After screening out identified peptides that had exact amino acid sequence matches in the human proteome (treating leucine and isoleucine as identical matches), an average of 339.4 PSMs matching an average of 169.8 peptides in 159.4 proteins were identified in the samples from *O. volvulus*-positive individuals, compared to an average of 114.7 PSMs, 72.8 peptides, and 70.4 proteins in the samples from uninfected individuals (false positives; Table 1). In the whole-worm lysate sample from adult female *O. volvulus*, 70,226 PSMs identified 21,109 peptides in 2405 proteins. Data from this sample were analyzed in order to identify proteins, peptides, and relative abundance (quantified by PSMs) in adult female worms. This analysis provided additional confidence in results from human samples when the same peptides and PSMs were detected by MS in *O. volvulus*-positive plasma and urine samples. The PSM counts for every detected peptide in every sample are provided in supplemental Table S3.

We used genomic and extensive transcriptomic (including RNAseq profiles of individual adult females) databases to provide additional insights into putative protein functions and specificity of candidate peptides for *O. volvulus* (compared to human and to other parasitic nematodes co-endemic to *O. volvulus*) to assess each identified protein’s potential for use as a biomarker for adult *O. volvulus* infection. Our comprehensive functional annotation data ensure that the maximum number of proteins have useful descriptions for interpretation of results. We also utilized gene expression data from eight adult female *O. volvulus* samples (12) to identify genes that are consistently and highly expressed in adult females. We also evaluated the orthology of each protein by examining orthologous protein families spanning the phylogeny of nematodes and hosts and by quantifying protein similarity by BLAST searches to species of interest. All of this detailed annotation data are provided for all *O. volvulus* proteins in an MS Excel database saved as supplemental Table S4; we used this information to evaluate and prioritize a list of top-ranked proteins as described below.

### Evidence-Based Detection of Promising Biomarker Candidates

**Fig. 2:**
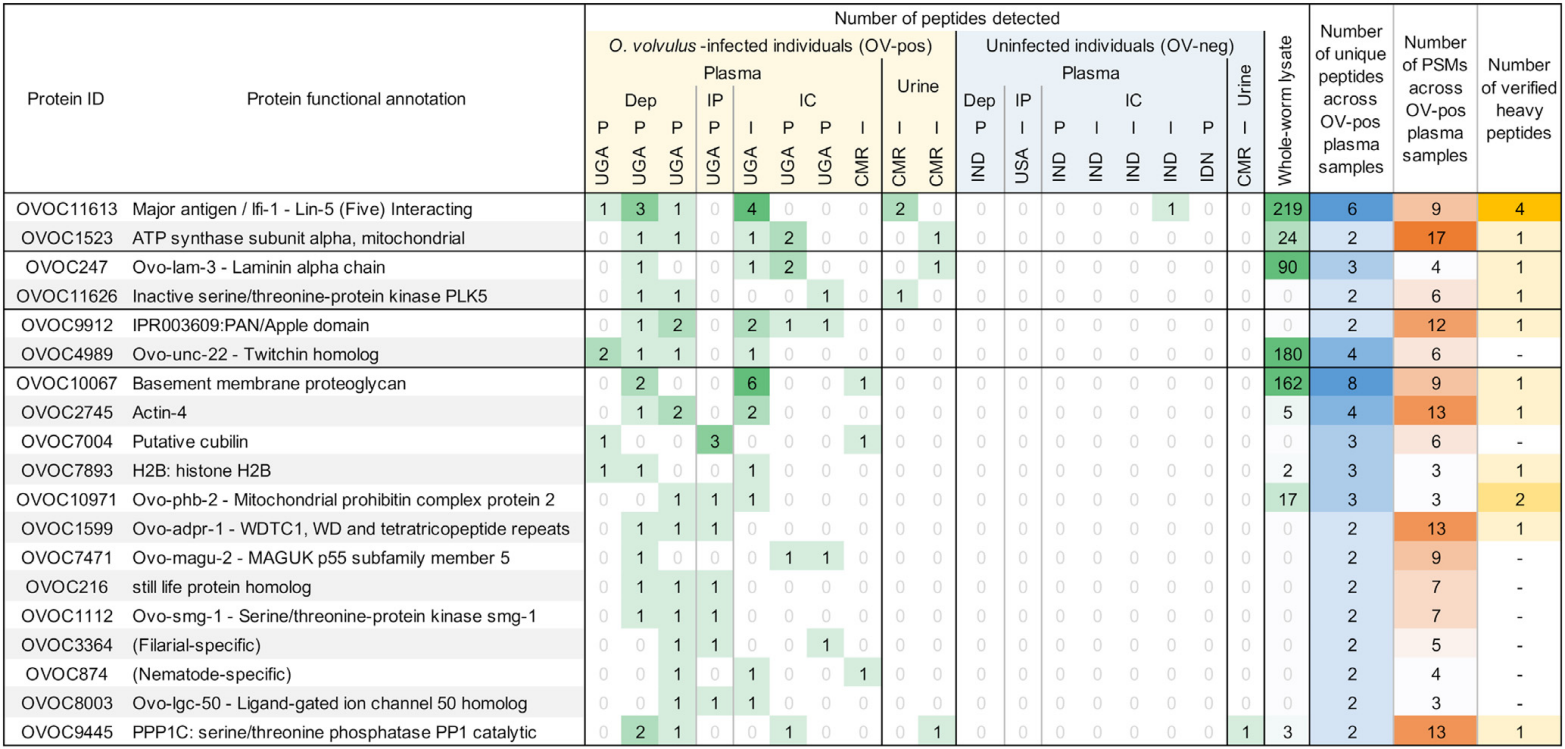
**A summary of results for the 19 high-priority protein targets identified according to the filtering criteria shown in**Fig. 1**.***Green numbers* represent the number of detected peptides in each sample, *blue numbers* represent the number of unique peptides from the protein that were identified in *O. volvulus*-positive plasma samples, and *orange numbers* represent the sum of peptide-spectral matches (PSMs) across all peptides in all *O. volvulus*-positive plasma samples. Proteins are annotated by assigned protein IDs, KEGG enzyme annotations, and InterPro functional domains. I = plasma/urine from individual person, P = pooled plasma from multiple individuals. Dep = depleted plasma, IP = immunoprecipitation from plasma samples, IC = immune complex from plasma samples.

High-priority *O. volvulus* biomarkers were those that were present in multiple samples from infected individuals (OV-pos), but not in samples from uninfected individuals (OV-neg). The overall workflow for the filtering and ranking of candidate protein biomarkers is shown in Fig. 1. Out of 12,224 total annotated *O. volvulus* proteins, 989 were detected across any of the OV-pos plasma samples, and 803 of those were not also detected in any OV-neg plasma samples (allowing up to one false-positive peptide match from a candidate biomarker protein from a single OV-neg sample as long as that peptide was not also detected in an OV-pos sample). Of these 803 proteins, 90 had two or more peptides detected across OV-pos samples. This requirement increased our confidence in protein identifications, and these 90 proteins were considered to represent the “true positive” protein set. Compared to the “false positive” set of 207 proteins with detection in OV-neg samples (not part of the 803 as defined in Fig. 1), the true positives (i) were more likely to be functionally annotated (36) (56.7% of true positives *versus* 41.1% of false positives, Fischer exact test *p* = 0.016); (ii) had much higher gene expression levels across the adult female RNA-seq dataset (average fragments per kilobase per million reads of 420.3 among true positives *versus* 49.0 in false positives, two-tailed *t* test *p* = 0.0098); and (iii) were more likely to be phylogenetically conserved across nematodes (55.5% of true positives *versus* 41.1% of false positives, Fisher exact test *p* = 0.023). Of these 90 “true positive” proteins, 19 were detected in at least three of the OV-pos plasma samples (Fig. 2). These 19 proteins were considered to be “promising biomarker candidates” that merit further study.

Confirmation of detected peptide sequences from the 19 promising biomarker candidates was performed by spectral matching to isotope-labeled synthetic peptides (“heavy peptides”). The MS/MS spectra from the heavy peptides were compared to the observed spectra of candidate biomarker peptides with regard to agreement to m/z values, intensities, and mass errors (supplemental Table S5). This analysis was used to confirm peptides for 15 peptides from 11 of the top-ranked 19 proteins (described below). This analysis was intended to provide an additional level of confidence for detected proteins. However, failure of this verification does not exclude proteins as potential biomarkers, because some peptides could not be successfully synthesized and because low concentrations of native biomarker in plasma samples can result in an insufficient number of fragment ions to align confidently confirm a match between synthetic heavy and naturally present peptides.

### Favorable Properties-Based Ranking of the 19 High-Priority Candidates

**Fig. 3:**
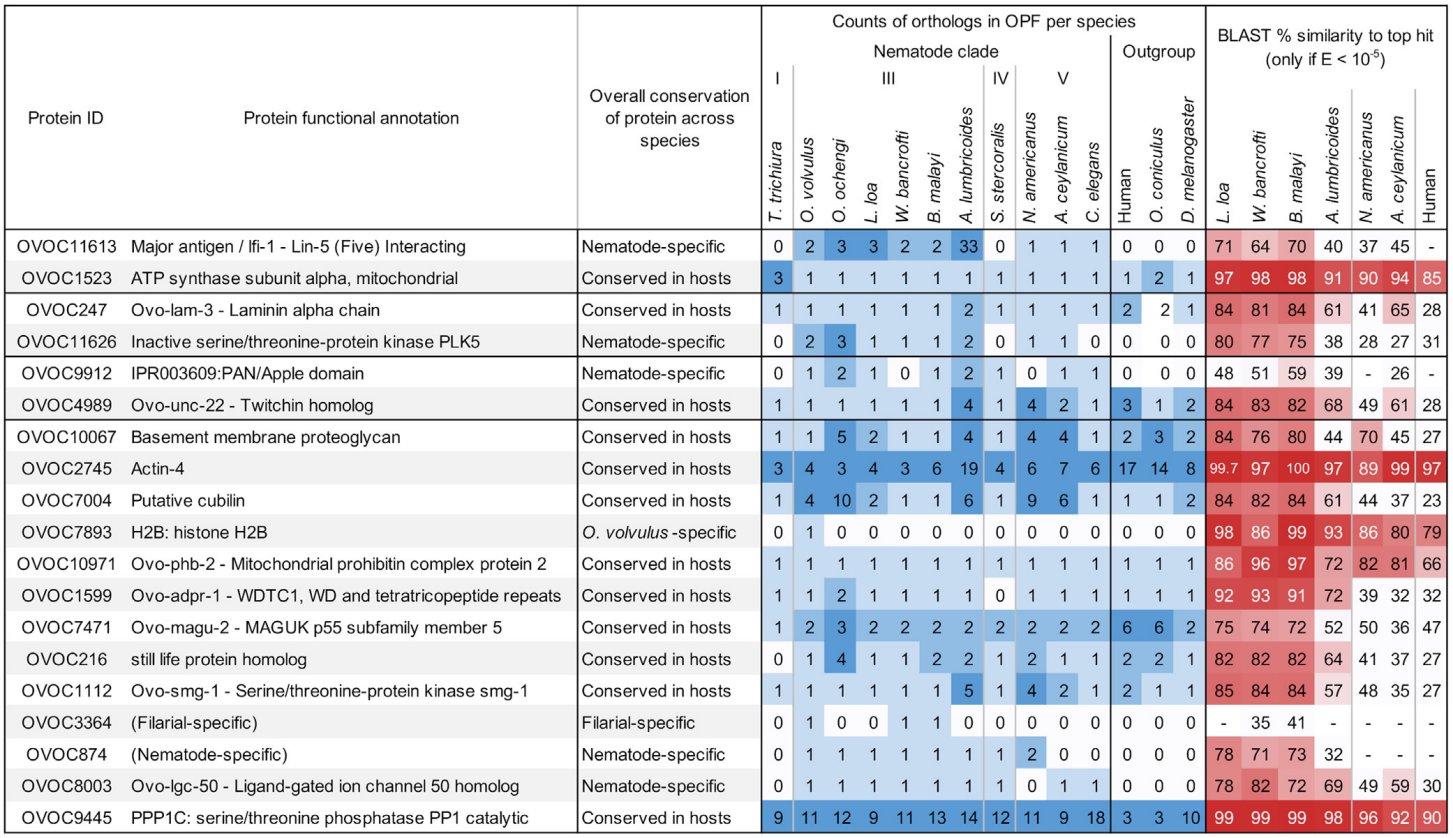
**Conservation of the 19 prioritized proteins based on Orthofinder results and BLAST amino acid sequence similarity of *O. volvulus* proteins to orthologous proteins in other relevant species**.

The 19 most promising biomarker candidates were subsequently ranked based on several properties (Fig. 1). First, the proteins identified in the highest number of OV-pos plasma samples were ranked at the top of the list, with four proteins being identified in four or more OV-pos plasma samples. Proteins present in the same number of samples were then ranked by the number of unique peptides that supported their detection and then based on the number of PSMs that supported their detection in different plasma samples (Fig. 2). Additional functional annotation data for each of the proteins are provided in Table 2, and phylogenetic conservation data are shown in Fig. 3. Functional annotation data include missense + nonsense single nucleotide polymorphism (SNP) rates for each protein across sequenced strains of *O. volvulus*, as identified in a previous study (46). Across all *O. volvulus* proteins, this average SNP rate was 5.06 SNPs/kb, so our top three candidates were all below average in terms of strain-to-strain sequence variation, and none of our top 19 candidates showed significant variability between forest and savannah strains or between West Africa and Ecuador strains of *O. volvulus* (46).

**Table 2:** Additional annotation and gene expression data for 19 prioritized biomarker candidate proteins

Protein ID	Protein functional annotation	Protein length (AA)	Predicted secretion	Gene exp. Level (avg. adult female FPKM)	Gene expression consistency (% of adult females)	Missense + nonsense SNP rate (Choi et al, 2016)
OVOC11613	Major antigen/lfi-1 - Lin-5 (Five) Interacting	2021	−	17.0	100%	3.6
OVOC1523	ATP synthase subunit alpha, mitochondrial	535	−	1963.9	100%	1.2
OVOC247	Ovo-lam-3 - Laminin alpha chain	3358	Signal peptide	24.9	100%	2.7
OVOC11626	Inactive serine/threonine-protein kinase PLK5	268	Nonclassical	2.0	100%	11.2
OVOC9912	IPR003609:PAN/Apple domain	628	Signal peptide	0.3	87.5%	15.4
OVOC4989	Ovo-unc-22 - Twitchin homolog	6785	−	28.9	100%	2.3
OVOC10067	Basement membrane proteoglycan	3415	Signal peptide	0.9	100%	7.1
OVOC2745	Actin-4	376	−	359.1	100%	0.9
OVOC7004	Putative cubilin	4112	Signal peptide	0.0	12.5%	22.0
OVOC7893	H2B: histone H2B	380	−	72.2	100%	2.6
OVOC10971	Ovo-phb-2 - Mitochondrial prohibitin complex protein 2	568	−	275.7	100%	4.1
OVOC1599	Ovo-adpr-1 - WDTC1, WD and tetratricopeptide repeats	597	−	21.8	100%	0.6
OVOC7471	Ovo-magu-2 - MAGUK p55 subfamily member 5	861	−	0.6	100%	8.1
OVOC216	still life protein homolog	987	−	12.8	100%	1.7
OVOC1112	Ovo-smg-1 - Serine/threonine-protein kinase smg-1	2527	−	13.8	100%	3.8
OVOC3364	(Filarial-specific)	166	Signal peptide	0.0	38%	12.5
OVOC874	(Nematode-specific)	239	−	106.1	100.0%	0.0
OVOC8003	Ovo-lgc-50 - Ligand-gated ion channel 50 homolog	221	−	0.1	63%	8.7
OVOC9445	PPP1C: serine/threonine phosphatase PP1 catalytic	671	−	9.8	100.0%	0.0

Included are the protein lengths (amino acids), predicted secretion, gene expression in adult females (RNA-seq), gene expression consistency among adult females, and the rate of missense + nonsense single nucleotide polymorphisms (SNPs) per kilobase of protein sequence, based on results from Choi et al, 2016 (46).

Abbreviation: FPKM, fragments per kilobase per million reads.

We identified four *O. volvulus* proteins in the OV-pos urine samples that were absent in the OV-neg urine sample. Urine results were used to corroborate plasma results, and urine testing was not part of the initial identification of the 19 high-priority candidates. Two of the proteins detected in urine were present in four or more OV-pos plasma samples (OVOC11613 and OVOC1523), and two were detected in three OV-pos plasma samples (OVOC247 and OVOC11626).

The top six proteins among the top 19 prioritized protein candidates (see bottom of Fig. 1) were the four supported by identification in the urine plus two additional proteins (OVOC9912 and OVOC4989) that were detected in four or more OV-pos plasma. These six proteins are described in greater detail below. It should be noted that one of the 19 prioritized biomarker candidates (OVOC9445) was also detected in an OV-pos urine sample. However, because one OV-neg urine sample contained one peptide from that protein (TFTDCFNCLPVAAIIDEK), the protein was ranked at the bottom of the list of 19 prioritized proteins. Below, additional details are provided for the top prioritized candidates, as well as other proteins of interest from the consistently detected and ranked list.

### Major Antigen/LFI1 (OVOC11613) Is the Biomarker Candidate with the Most Supporting Evidence

**Fig. 4:**
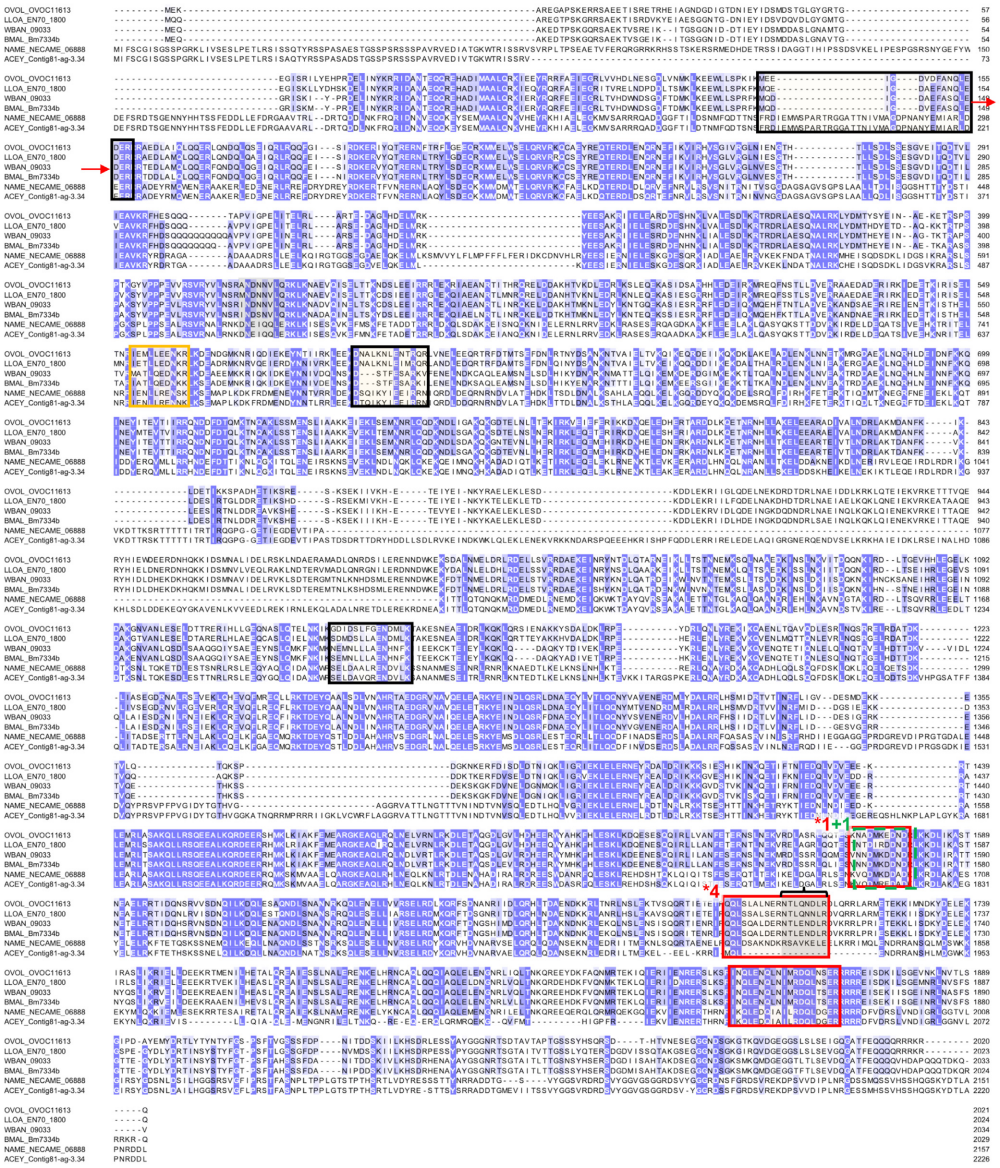
**Multiple protein alignment (M-Coffee) for Major Antigen (OVOC11613) and its most significant BLAST hits in *L. loa* (LLOA), *W. bancrofti* (WBAN), *B. malayi* (BMAL), *N. americanus* (NAME), and *A. ceylanicum* (ACEY).** Shades of *blue* indicate level of amino acid conservation. Peptides highlighted in *red* were detected in plasma samples from *O. volvulus* infected individuals, peptides highlighted in *black* were detected in plasma samples and verified by the heavy peptide spectral matching, peptides highlighted in *green* were detected in urine samples from *O. volvulus* infected individuals, and the peptide highlighted in *orange* was detected in urine and verified by the heavy peptide spectral matching. The sequence marked with a *black bracket* was a peptide also identified for Major Antigen protein in *Onchocerca lupi* (Latrofa *et al*, 2021). ∗Numbers shown indicate that a peptide was detected in multiple samples, red = plasma, green = urine.

OVOC11613 was identified as the top prioritized candidate because (i) peptides from it were detected in four OV-pos plasma samples with a total of six unique peptides including as many as four peptides in a single OV-pos sample (Fig. 2); (ii) two peptides supported its identification in an OV-pos urine sample: IEMLLEENKR (verified by heavy peptide spectral matching) and NADMKEDNDR (also detected in OV-pos plasma samples and both of which were not identically matched in other filarial nematode species; Fig. 4); (iii) it is nematode-specific, sharing no significant homology with human protein, either by orthologous protein grouping or by BLAST (Fig. 3); (iv) it had moderate but consistent gene expression in individual adult *O. volvulus* females; Table 2); (v) four of the detected peptides were confirmed by the synthetic heavy peptide spectral matching, and (vi) it has a very close ortholog in *Onchocerca lupi* (Ol-MJA, “Major antigen”, as annotated by UniProt (47), P21249) that was identified as a promising target for canine antibody serology in in a prior study (48). Additionally, the *O. ochengi* OVOC11613 ortholog was the third most abundant protein detected in adult female worms in a previous mass spectrometry study (49). While one peptide (out of six) from this protein was detected in one OV-neg plasma sample, the peptide sequence was only supported by a single PSM. Since that peptide was not detected in any of the OV-pos samples, it did not eliminate the protein in the filtering process (since this single detection could be an artifact/false positive). In addition, OVOC11613 was detected by 219 unique peptides in the *O. volvulus* lysate sample, suggesting that it is a highly abundant protein in adult worms and further supporting its adult *O. volvulus* origin in the plasma and urine.

OVOC11613 was called a “major antigen” based on its reactivity with antibodies in sera from humans with onchocerciasis (50). A subsequent study confirmed that the protein was expressed in adult female *O. volvulus* worms (51). In the recent *O. lupi* study (48), an immunoblotting/nLC-MS/MS proteomics approach was used to identify *O. lupi* proteins that were immunoreactive with antibodies in plasma from infected but not uninfected dogs. One peptide for OVOC11613 from that study (QQLSLALNERNTLQNDLR) was also identified here in three different OV-pos–depleted plasma samples, and one OV-pos IC plasma sample. This *O. volvulus* peptide has at least three amino acid sequence differences relative to related peptides in other filarial parasite species that infect humans. These features highlight the potential for this peptide as a biomarker candidate and show that it warrants further evaluation.

The biological function of OVOC11613/major antigen has not been described, and no primary sequence similarity was detected to any protein domain(s) or proteins in InterPro (39) or KEGG (41). Its top BLAST match in *C. elegans* was to “LFI1, Lin-5 interacting protein” (ZC8.4), which is involved in spindle formation (52), but this had very low overall sequence similarity to OVOC11613 (62.5% dissimilarity over 80% of the protein length).

### OVOC1523/ATP Synthase Alpha Was the Second-Ranked Overall Candidate

**Fig. 5:**
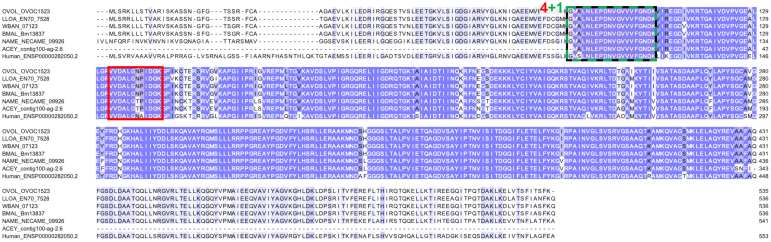
**Multiple protein alignment (M-Coffee) for ATP synthase subunit alpha (OVOC1523) and its most significant BLAST hits in *L. loa* (LLOA), *W. bancrofti* (WBAN), *B. malayi* (BMAL), *N. americanus* (NAME), *A. ceylanicum* (ACEY), and human.** Shades of *blue* indicate level of amino acid conservation. Peptides highlighted in *red* were detected in plasma samples from *O. volvulus* infected individuals, peptides highlighted in *black* were detected in plasma samples and verified by the heavy peptide spectral matching, and peptides highlighted in *green* were detected in urine samples from *O. volvulus* infected individuals. ∗Numbers shown indicate that a peptide was detected in multiple samples, red = plasma, green = urine.

OVOC1523 (ATP synthase subunit alpha, mitochondrial homolog) was prioritized because (i) it was identified with two unique peptides in four OV-pos plasma samples (two depleted plasma samples and two IC preparations) (Fig. 2); (ii) it was identified in an OV-pos urine sample by the peptide GMALNLEPDNVGVVVFGNDK, which was also detected in all four plasma samples that contained this protein (Fig. 5). The presence of that peptide was confirmed by the synthetic heavy peptide spectral matching, and (iii) it was more highly expressed than 99.67% of all genes in adult female *O. volvulus*. Although the *C. elegans* ortholog of OVOC1523 (ATP-1, H28O16.1a) functions as an ATP synthase in mitochondria, it also has an independent function responsible for iron acquisition in nematodes (53). An additional 24 unique peptides supported the identification of this protein in *O. volvulus* adult worm lysate. Although OVOC1523 is broadly conserved in nematodes and mammals (Fig. 3), it has 15% sequence dissimilarity to the closest human protein ortholog (ATP5F1A). Because some of the dissimilar sequences include portions of the molecule detected by MS in human samples, the protein has some potential as a biomarker for *O. volvulus* infection.

### OVOC247 and OVOC11626 Were Detected in Both the OV-Pos Plasma and Urine Samples

**Fig. 6:**
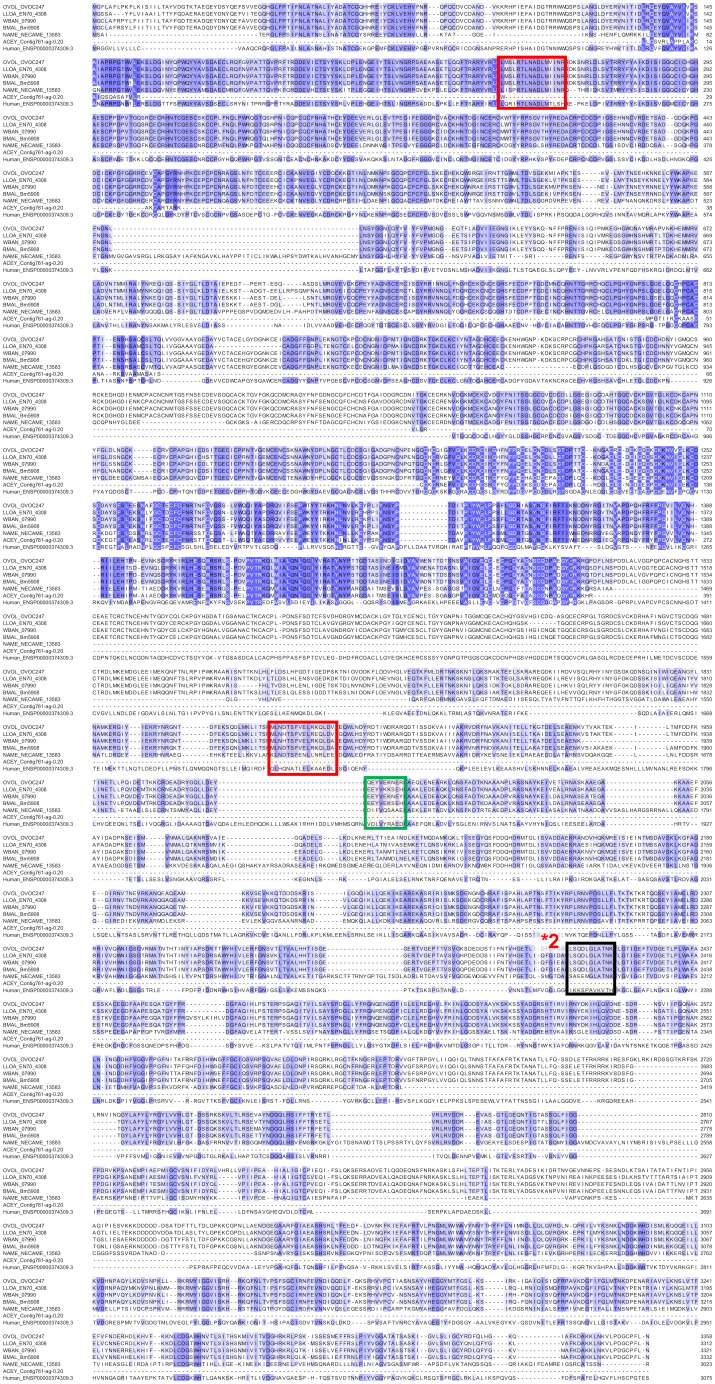
**Multiple protein alignment (M-Coffee) for laminin alpha chain (OVOC247) and its most significant BLAST hits in *L. loa* (LLOA), *W. bancrofti* (WBAN), *B. malayi* (BMAL), *N. americanus* (NAME), *A. ceylanicum* (ACEY), and human**. Shades of *blue* indicate level of amino acid conservation. Peptides highlighted in *red* were detected in plasma samples from *O. volvulus* infected individuals, peptides highlighted in *black* were detected in plasma samples and verified by the heavy peptide spectral matching, and peptides highlighted in *green* were detected in urine samples from *O. volvulus* infected individuals. ∗Numbers shown indicate that a peptide was detected in multiple samples, red = plasma, green = urine.

OVOC247/laminin alpha chain was ranked third overall because (i) it was detected by three peptides in three OV-pos plasma samples (one depleted plasma, two IC); (ii) it was identified in an OV-pos urine sample by the peptide QEYVEKNER (Figs. 2 and 6); (iii) it has consistent and moderately high expression in adult female worms, (iv) it contains a signal peptide for secretion which increases the likelihood that viable adult worms might release the protein into the host; (v) it was highly abundant in whole-worm lysate (with 90 detected peptides), and (vi) one peptide from OVOC247 was confirmed by synthetic heavy peptide spectral matching. Although it has an ortholog in human hosts, its human ortholog (LAMA1) only has 27.7% sequence similarity. Nematode laminins are glycoproteins that form a major polymer within basement membranes, and they are crucial for organizing extracellular matrix (54), which may explain why they would be identified in the plasma and urine.

**Fig. 7:**
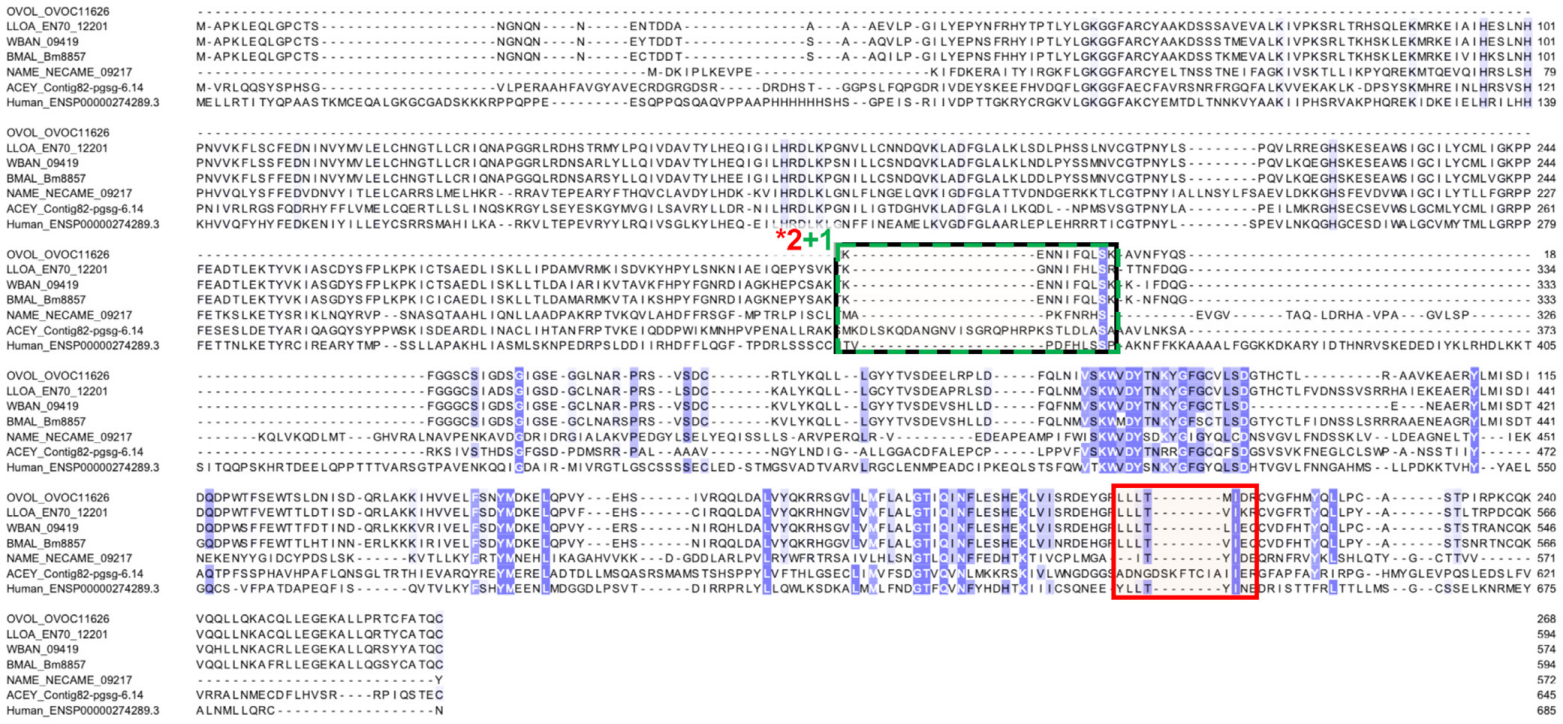
**Multiple protein alignment (M-Coffee) for PLK-5 (OVOC11626) and its most significant BLAST hits in *L. loa* (LLOA), *W. bancrofti* (WBAN), *B. malayi* (BMAL), *N. americanus* (NAME), *A. ceylanicum* (ACEY), and human.** Shades of *blue* indicate level of amino acid conservation. Peptides highlighted in *red* were detected in plasma samples from *O. volvulus* infected individuals, and peptides highlighted in *green* were detected in urine samples from *O. volvulus* infected individuals. ∗Numbers shown indicate that a peptide was detected in multiple samples.

OVOC11626/PLK5 (inactive serine/threonine-protein kinase) was highly ranked because (i) it was identified by three unique peptides that were detected in four different OV-pos plasma samples (three depleted, one IC; Fig. 2); (ii) it was identified in an OV-pos urine sample by the peptide KENNIFQLSK, which was also detected in two of the depleted plasma samples and was supported by the synthetic heavy peptide spectral matching (Fig. 7); (iii) it was nematode-specific by the OrthoFinder classification, with only 31% similarity to any human protein (Fig. 3); (iv) it was predicted to be non-classically secreted, and (v) it had consistent expression across all adult females in the RNA-seq dataset (Table 2). PLK5 has weak homology with *C. elegans* PLK2 (28% similarity) which is involved in crossover during meiosis (55), but the function of PLK5 in *O. volvulus* is unknown.

### OVOC9912 and OVOC4989 Were Identified in More Than Three OV-Pos Plasma Samples

OVOC9912 (PAN/apple domain) was the only protein other than the top prioritized candidate (OVOC11613) to be identified in five different OV-pos plasma samples (two depleted and three IC samples), and one of the detected peptides was confirmed by synthetic heavy peptide spectral matching. It is nematode-specific, with no orthology to any human protein, and has the lowest orthology of the top six proteins with other parasitic nematodes that infect humans (Fig. 3), making it an attractive biomarker candidate due to its *O. volvulus*-specificity. It contains a predicted signal peptide, suggesting that it is secreted by living worms, which might explain its relatively consistent detection in human samples despite its low gene expression in RNA-seq datasets. OVOC9912 lacks detailed functional annotation, with the only functional evidence being a “PAN/Apple domain” (InterPro domain annotation, IPR003609), a domain which is commonly found in plasminogen proteins as well as in nematode proteins (56).

OVOC4989 (unc-22, twitchin homolog) is the last of the 19 candidates to be detected in more than three OV-pos plasma samples (three depleted, one IC). Four unique peptides were detected in plasma samples. This protein was highly abundant in the worm lysate sample (180 peptides), and it has low conservation with human proteins. Nematode unc-22 is involved in myosin functional regulation in muscle tissue (57). It is an extremely large protein (6785 amino acids in the current OVOC4989 protein model), so its presence in the blood may be due to its high relative abundance in the *O. volvulus* muscle tissue.

### Additional Candidates of Interest

OVOC10067 (basement membrane proteoglycan) was identified here by eight unique peptides across three OV-pos plasma samples (one depleted, two IC), and it was identified as one of 33 serodiagnostic candidate proteins in our prior proteomics study of *O. volvulus* proteins that were bound by antibodies present in plasma from onchocerciasis patients (12). One of its supporting peptides in human plasma was confirmed by the synthetic heavy peptide spectral matching, and this protein also contains a signal peptide sequence. Additionally, the *O. ochengi* OVOC10067 ortholog was the second-most abundant protein detected in adult female worms and was highly abundant in *O. ochengi* nodule fluid in a previous mass spectrometry study (49). Thus, this candidate, although not detected in our pilot studies with urine, is an attractive target for an adult female-specific biomarker.

OVOC2745 (actin-4) was supported by four peptides across three OV-pos plasma samples (one confirmed by synthetic heavy peptide spectral matching), and it was also identified as an antibody target in the canine *O. lupi* study (48). It had high and consistent gene expression in adult female *O. volvulus.* However, this protein has high homology (97.3%) with the human protein ACTG1, and its function as a structural protein reduces its potential as biomarker candidate for detection by immunoassay.

Overall, all of the proteins shown in Fig. 2 are supported by the proteomics evidence, but some candidates such as OVOC7004, OVOC3364, and OVOC8003 have lower RNA-seq expression and are inconsistently expressed across adult female worm RNA-seq samples (Table 2). All of the protein properties presented in Figs. 2 and 3, Table 2 and supplemental Table S4 should be considered in future evaluations of these biomarker candidates.

## Conclusions

The direct proteomics discovery approach utilized here facilitated the discovery of a short list of prioritized potential novel biomarkers with a relatively high rate of true positivity, with some candidates detected in multiple samples from persons with onchocerciasis. This is the first report of regular and deep scale MS proteomics being used to directly screen human plasma and urine samples for parasite proteins that might serve as biomarkers for active *O. volvulus* infection. Other studies have focused on identifying *O. volvulus* proteins for use as targets for antibody assays (*e.g.*, (6, 7)) or on the prioritization of identified potential biomarkers based on an analysis of gene expression profiles or other bioinformatics data (*e.g.*, (12)). However, this type of evidence only indicates detection of potential circulating antigens and does not quantify direct detection in the blood or urine, as performed here. Direct detection has been identified for *O. volvulus*-originated metabolites (*e.g.*, (14, 17)) and phospholipids (18), but the consistent detection of small amounts of metabolites and phospholipids among all the metabolites found in human blood and urine is very difficult to achieve, and they may also be conserved among other endemic helminths. Additional validation of the potential biomarkers presented here is provided by their absence of detection in uninfected samples, and the manual validation of spiked-in heavy peptides to confirm the proteomic detection of key peptides.

There have been *Onchocerca* biomarkers proposed in the past which have later failed validation, and likewise, additional work will be needed to assess the sensitivity and specificity of the biomarker candidates identified in this study. This will require the development of sensitive assays and the testing of large panels of positive and negative samples. The lack of available genome data for the co-endemic filarial nematode *Mansonella* spp. necessitates future screening to ensure that biomarker peptide sequences are not conserved with this species, ideally by testing individuals infected only with *Mansonella*. Likewise, the testing of *O. volvulus*-infected individuals treated with ivermectin who are negative for the skin snip test would support the validation of the biomarkers as being derived from the adult females and not being from microfilariae. It is possible that multiplex assays for multiple biomarkers will be required to achieve high sensitivity, and the top 19 candidates are presented here in order to provide the maximum potential for a functional test, with the assumption that many will not work in a final biomarker test. Overall, this study has identified a promising novel set of proteins that will be carried forward to develop assays that can be used for diagnosis of *O. volvulus* infections and for assessing the efficacy of new treatments with samples from clinical trials. Such a test would be invaluable for screening individuals who currently test negative in traditional skin snip tests, but who still harbor live adult *O. volvulus* worms, necessitating further treatments in the area which currently may not be administered.

## Data Availability

The MS proteomics data have been deposited to the ProteomeXchange Consortium (http://proteomecentral.proteomexchange.org) *via* the iProX partner repository with the data set identifier PXD033659. Proteomics sample metadata and accession IDs per sample are provided in supplemental Table S1. Machine setting parameters for proteomics runs including QE, EASYnLC, timsTOF, and Nanoelute, as well as MaxQuant configuration parameters are provided in supplemental Table S2. Peptide-spectral match counts for each peptide across each sample, including precursor charge, mass/charge, modifications observed, and scores are provided in supplemental Table S3. Detected peptide counts per sample, complete functional annotation data, orthology information, and gene expression data for all *O. volvulus* proteins are provided in supplemental Table S4. Output from the isotope-labeled standard peptide (heavy peptide) fragment ion spectral matching analysis for all confirmed proteins is provided in supplemental Table S5.

## Supplemental data

This article contains supplemental data (12, 46).

## Conflicts of interest

The authors declare no competing interests.
